# *In Silico* Modeling and Functional Interpretations of Cry1Ab15 Toxin from *Bacillus thuringiensis* BtB-Hm-16

**DOI:** 10.1155/2013/471636

**Published:** 2013-10-22

**Authors:** Sudhanshu Kashyap

**Affiliations:** National Bureau of Agriculturally Important Microorganisms (ICAR), Kusmaur, Kaithauli, Maunath Bhanjan, Uttar Pradesh 275101, India

## Abstract

The theoretical homology based structural model of Cry1Ab15 **δ**-endotoxin produced by *Bacillus thuringiensis* BtB-Hm-16 was predicted using the Cry1Aa template (resolution 2.25 Å). The Cry1Ab15 resembles the template structure by sharing a common three-domain extending conformation structure responsible for pore-forming and specificity determination. The novel structural differences found are the presence of **β**0 and **α**3, and the absence of **α**7b, **β**1a, **α**10a, **α**10b, **β**12, and **α**11a while **α**9 is located spatially downstream. Validation by SUPERPOSE and with the use of PROCHECK program showed folding of 98% of modeled residues in a favourable and stable orientation with a total energy *Z*-score of −6.56; the constructed model has an RMSD of only 1.15 Å. These increments of 3D structure information will be helpful in the design of domain swapping experiments aimed at improving toxicity and will help in elucidating the common mechanism of toxin action.

## 1. Introduction

*Bacillus thuringiensis *(Bt) a soil bacterium produces pertinacious toxin generally referred to as insecticidal crystal protein. This toxin belongs to a large family with target spectrum spanning insects, nematodes, flatworm, and protozoa [1]. In nature, Cry toxins are produced as crystalline protoxin (hence named Cry protein) within Bt sporangia, and after ingestion by a susceptible insect larva, these protoxins are solubilized and proteolytically cleaved into an active toxin fragment that binds to at least one of the four different types of high affinity receptors and later get inserted into the brush border epithelium. The insertion of toxin creates pores in the cell membrane that causes the leaching of the cellular electrolytes. This disruption causes cell lyses and finally larval death [2]. So far, Cry1 toxins have extensively been used in studies of insect control either as transgenic spores or as spray formulations. Where three-dimensional crystal structure of Cry1 family of protein is concerned, few of the toxins in solutions have been analysed by X-ray diffraction crystallography [3–9], and a few of them have been predicted using the homology modelling method [10–12]. All these toxins have a different toxicity spectrum; in spite of this, these proteins show a similar tertiary organization. This property impels for elucidation of three-dimensional structures of the rest of the reported Cry1 family members for possible setting down of unifying mechanisms underlying the toxicity. There are currently many templates for protein structures prediction available from the Protein Data Bank (PDB) [13], and all such templates are constantly increasing in number. For three-dimensional structure prediction, modeled structure, by applying template-based modeling, has become so accurate that they can be applied for molecular replacement information in many cases. Therefore, in this paper, as an increment in a structure elucidation, the model of the Cry1Ab15 toxin is reported based on the hypothesis of structural similarity with Cry1Aa toxin [7]. This model supports existing hypotheses of receptor insertion and will further provide an initiation point for the domain-swapping and mutagenesis experiments among different Cry toxins.

## 2. Materials and Methods

### 2.1. Sequence Data

The amino acid sequence of the putative Cry1Ab15 protein of *Bacillus thuringiensis *was retrieved from the National Center for Biotechnology Information (NCBI) database. The sequence accession number was AAO13302. It was ascertained that the three-dimensional structure of the protein was not available in the Protein Data Bank; hence, the present exercise of developing the three-dimensional model was undertaken.

### 2.2. Template Selection and Structure Prediction

Homology method-dependent modeling is an effective approach for a three-dimensional structure of protein provided by an experimentally obtained three-dimensional structure of homologous protein. All experimentally determined homologous protein can serve as a template for modeling. Since template selection is an important factor that affects quality, therefore, an attempt was made for a suitable template searching using mGenTHREADER [14], which is an online tool for searching similar sequences, based on sequence and structure-wise similarity. The target protein was 577 amino acid long stretches. From the homologous searching, Cry1Aa (PDB: 1CIY, resolution 2.25 Å) was selected as a template protein. Finally, amino acid sequence alignment between the target (Cry1Ab15) and template protein was derived using the MEGA4 software [15]. The three-dimensional structure of target protein was predicted by using the alignment file in MODELLER software [16] whereby predicted structure was returned.

### 2.3. Homology Modeling of Cry1Ab15

**Figure 1:**
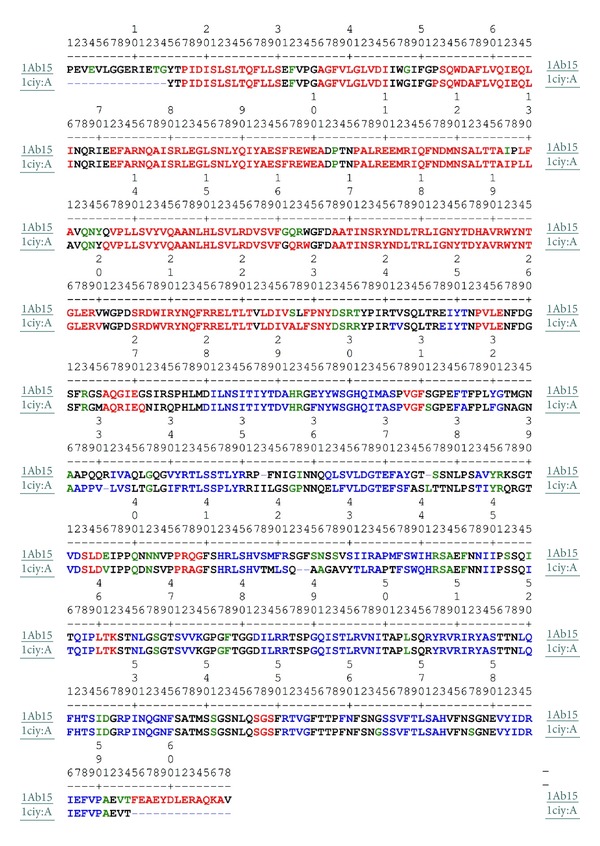
Amino acid sequence alignment of the Cry1Ab15 with Cry1Aa (1ciy: A). The residues highlighted in red color represent helix; those in blue represent strand; in green represent turn; and those in black represent coil, and alignment is generated using SAS software.

The possible outliers and side chains static constrain refinement of the developed model was performed on Summa Lab server [17] after the selected theoretical model were further subjected to a series of tests for evaluating its consistency and reliability. Backbone confirmation was evaluated by the inspection of the Psi/Phi Ramachandran plot from RAMPAGE web server [18]. The energy criterion was evaluated by ProSA web server [19], which compares the potential of mean forces derived from a large set of NMR and X-ray crystallographically derived protein structures of similar sizes. Potential deviations were calculated by SUPERPOSE web server [20] for root mean square deviations (RMSD) between target and template protein structure. The comparative analysis of generated model showed it to be superimposable. The secondary structure visualization was made using PDBsum [21], and amino acid sequence alignments are generated with SAS software [22] (Figure 1). The visualization of models was performed on UCF Chimera software [23] and PyMOL [24] loaded on a personal computer machine that has an Intel Quad core processor and four gigabytes of random accessed memory. Figures and electrostatic potential calculations were generated with PyMOL0.99rc6. The final model was submitted to the PMDB database [25] to obtain protein model databank (PMDB) identifier PM0076556.

## 3. Results and Discussion

**Figure 2:**
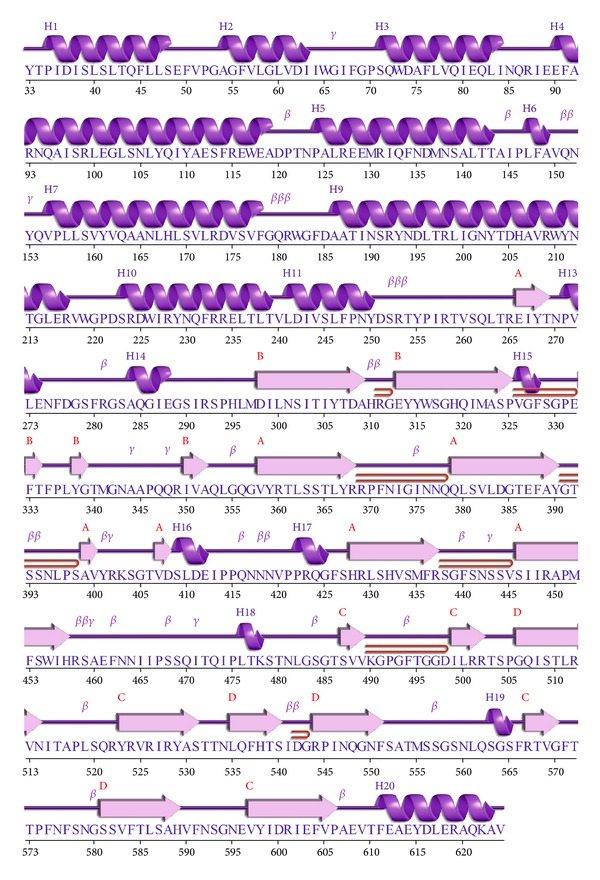
The two-dimensional structure annotation showing sequential arrangements of helices and sheets in Cry1Ab15 toxin molecule using the PDB Sum (http://www.ebi.ac.uk/pdbsum/). The structure is as the spiral shape are helix labeled as H1 and H2; and the arrows as strands are labeled by their sheets A and B while motifs *β* are beta turn and *γ* are gamma turn while the bend tube shape is a beta hairpin.

**Figure 3:**
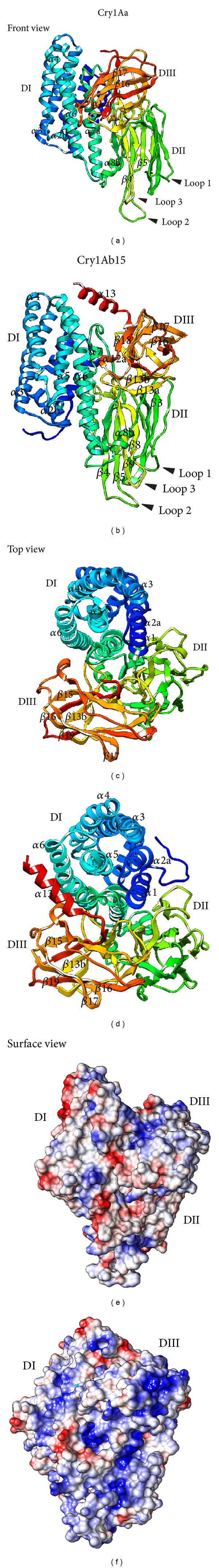
The comparative three-dimensional, three-domain structure of the Cry1Ab15 ((b), (d), (f)) and Cry1Aa ((a), (c), (e)) molecules.

**Figure 4:**
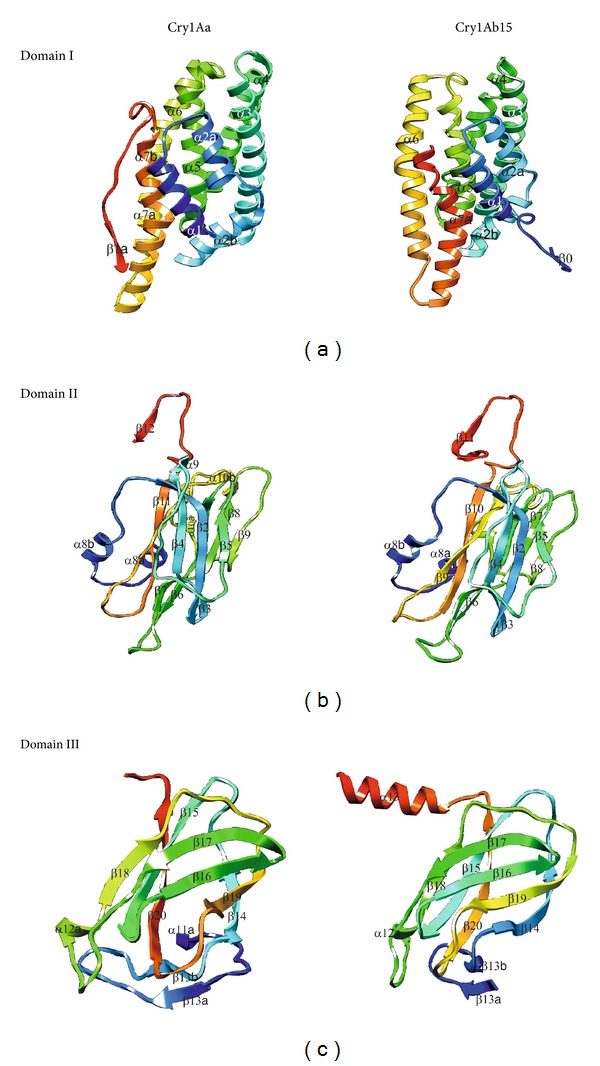
The comparative figures separately showing details among three structural domains of Cry1Ab15 and Cry1Aa molecules.

Sequence alignment showed 88.3% identity (Smith Waterman Score-3356; *Z*-Score-3981.3; *E* Value-6.4e-215) between the Cry1Ab15 and Cry1Aa. It is observed that a model tends to be reliable if identity percentage between the template and target protein is above 40%. Low degree of reliability arises when identity decreases below 20% [26]. Identity difference in the present case is sufficiently high to carry out the theoretical modeling for the Cry1Ab15 toxin stretch of 84–661 residues (Figure 1). Sequence alignment of domain I, domain II, and domain III was straightforward within the possible limits of flanking domains. Domain III is quite well conserved both on the N-terminal and C-terminal sides. Domain I is composed of residues 86–341 and consists of 9 *α*-helices and too small *β*-strands. All the helices in the Cry1Ab15 model were slightly longer than those in Cry1Aa (Table 1). The amphiphilicity (Hoops and Woods) values indicated an exposed nature of a few of the helices of domain I (*α*1, *α*2a, *α*2b, *α*3, and *α*6). These values correspond well with the accessibility calculated with Swiss PDB, except for *α*1, which is packed against domain II (Figure 2). It is possible that this helix will have some mobility, with an emphasis that one of the cutting sites by gut proteases is located close to the middle of this helix [27]. On the other hand, membrane insertion and pore formation are thought to occur through elements of domain I, composed of a bundle of six amphipathic *α*-helices surrounding the highly hydrophobic helix *α*5 [7]. Spectroscopic studies with synthetic peptides corresponding to domain I helices revealed that *α*4 and *α*5 have the greatest propensity for insertion into artificial membranes, although insertion and pore formation were more efficient when *α*4 and *α*5 were connected by a segment analogous to the *α*4-*α*5 loop of the toxin [28, 29]. A particularly large number of single-site mutations with altered amino acids from these helices, which lead to a strong reduction in the toxicity and pore-forming ability of the toxin, have been characterized [30–33]. Also, a site-directed chemical modification study has provided strong evidence that *α*4 lines the lumens of the pores formed by the toxin [34]. Recent studies have established that toxin activity is especially sensitive to modifications not only in the charged residues of *α*4 [33] but also in most of its hydrophilic residue [30]. Furthermore, the loss of activity of most of these mutants did not result from an altered selectivity or the size of the pores, but from a reduced pore-forming capacity of the toxin [34]. The charge distribution pattern in the Cry1Ab15 theoretical model corresponds to a negatively charged patch along *β*4 and *β*13 (Figures 3 and 4) of domains II and III, respectively. The Cry1Ab15 domain I model relates well with the data from Gerber and Shai [29] who have suggested that *α*4 and *α*5 insert into the membrane in an antiparallel manner as a helical hairpin. It is possible that according to the surface electrostatic potential of helices 4 and 5 there was a neutral region in the middle of the helices which probably indicates, if we follow the umbrella model and consider it to be correct, that both helices cross the membrane with their polar sides exposed to the solvent as it has been suggested by the results of mutagenesis experiments done by Girard et al. [31] with the Cry1Ac toxin. This region is also the most conserved among the Cry toxins. Girard et al. [31] demonstrated that mutations in the base of helix 3 and the loop between *α*3 and *α*4 that cause alterations in the balance of negative charged residues may cause loss of toxicity. Mutations in helices *α*2 and *α*6 and the surface residues of *α*3 have no important effect on toxicity; meanwhile, helices *α*4 and *α*5 seem to be very sensitive to mutations. Helix *α*1 probably does not play an important part in toxin activity after the cleavage of the protoxin. It is possible that the mutations aimed to an increasing the amphiphilicity in these helices will improve the pore-forming activity of the Cry1Ab15 type toxins. The structure of domain I of the toxin, the effect of site-directed mutagenesis in this domain on toxin activity, and the studies with hybrid toxins [35–37] all suggest that domain I, or parts of it, inserts 125 into the membrane and forms a pore. This idea is further supported by studies that show that truncated proteins corresponding to domain I of CryIA(c) [38] *δ*-endotoxin form ion channels in model lipid membranes similar to those formed by the intact toxins. After receptor binding, the network of contacts between *α*7, the helix in the interface between the pore-forming domain and the receptor-binding domain, and *α*5, *α*6, and, presumably, *α*4 helices may assist at the insertion of the *α*4-*α*5 hairpin into the membrane by the unpacking of the helical bundle that exists in the nonmembrane-bound form of the toxin. This hypothesis might account for the observation that *α*7 mutants are susceptible to proteolysis by either trypsin or midgut juice [39]. Our model also supports the notion that the *α*4-*α*5 hairpin is the major structural component in the lining of the pores formed by *δ*-endotoxin. Therefore, it is possible to create toxin variants with better membrane permeability potential by stabilizing the hairpin antiparallel structure by cross-linking *α*4 with *α*5. This postulation is important because mutations within transmembrane segments of proteins usually decrease or have no effect on the biological activities of these proteins. Thus, it is conceivable that the introduction of several salt bridges or other bonds between *α*4-*α*5 helices or the stabilization of the *α*4-*α*5 hairpin by the creation of bridging interactions between the *α*3-*α*4 and *α*5-*α*6 loops may result in a significantly enhanced toxic activity. Other studies also support the umbrella-like model for domain I insertion into membranes [34, 40, 41]. As for other Cry toxins, domain II of the Cry1Ab15 toxin consists of three Greek key beta sheets arranged in a beta prism topology. It is comprised of residues 350–508, one helix (*α*8), and 11 *β*-strands (Table 1). In the case of the three domain Cry toxins, specificity is mostly attributed to their capacity to bind to certain proteins located on the surface of the intestinal membrane through specific segments of domains II and III, composed mainly of *β* sheets [42, 43]. Loop *β*4-*β*5 is mostly hydrophilic, and the charged residues located at the tip of the loop are probably important determinants of insect specificity. As in loop *β*2-*β*3, few glycine residues are also present before a negatively charged residue supporting the hypothesis that correct orientation of charged residues in the specificity loops could be important in receptor recognition. Mutations in defined regions of the Cry1Aa toxin have identified residues 365–371 (equivalent to residues in the Cry1Ab15 *β*6-*β*7 loop) as essential for binding to the membrane of midgut cells of *Bombyx mori* [35, 44]. In the Cry1Ab15 model, this region is shorter than their counterparts in Cry1Aa. Loop *β*2-*β*3 seems also to be able to modulate the toxicity and specificity of Cry1C [45]. The dual specificity of Cry2Aa for Lepidoptera and Diptera has been mapped to residues 307–382 that corresponds in the Cry1Ab15 theoretical model to sheet 1, strand *β*6, and loop *β*6-*β*7. Domain III comprised residues 471–608 and showed high conservation of residues and the only important modification is a 3-residue deletion between *β*16 and *β*17. Several studies indicate that site mutations in conserve blocks reduce toxicity and alter channel properties at least in Cry1Ac [7] and Cry1Aa [42, 46], and divergence in block 5 element [8, 41] postulates an alternative mechanism of membrane permeabilization. 

**Figure 5:**
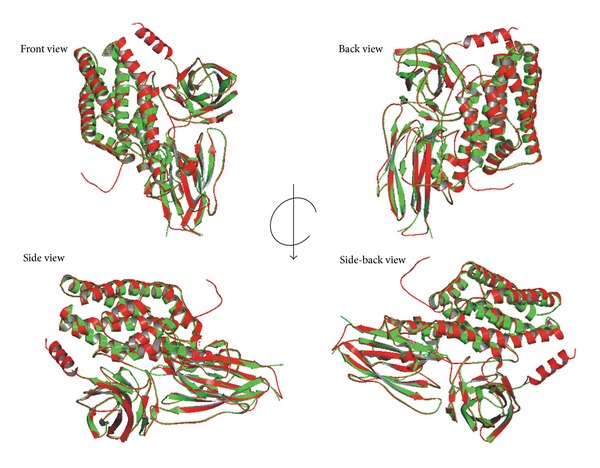
Superimposed backbone 3D structure between Cry1Aa1 (green) and Cry1Ab15 (red) coordinates. The RMSD for backbone and alpha carbons is 1.15. The image was generated using the SUPERPOSE software (http://wishart.biology.ualberta.ca/SuperPose/).

**Figure 6:**
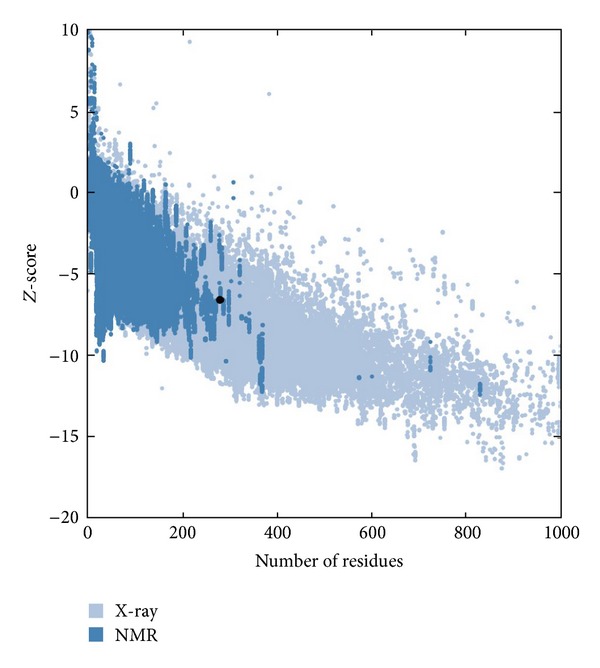
Evaluation of Cry1Ab15 using ProSA server (https://prosa.services.came.sbg.ac.at/). The plot indicating nearness of constructed structure with the native structures. The *Z*-score of evaluated model was −6.56, shown as a large black dot.

**Figure 7:**
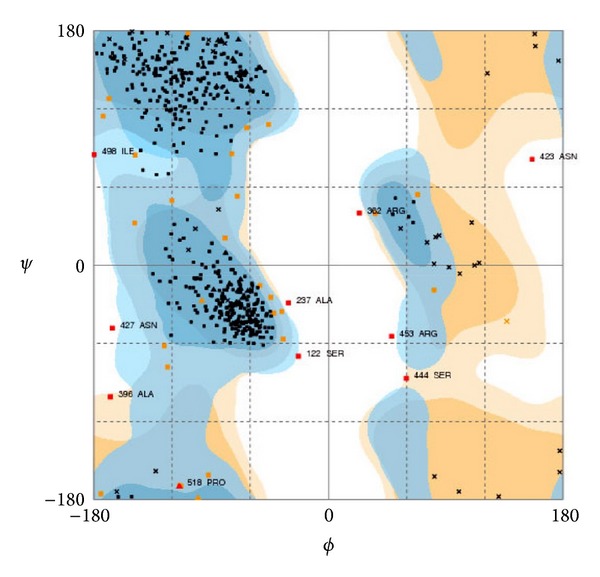
Ramachandran plot analysis of the Cry1Ab15 toxin oligomer showing placement of residues in deduced model. General plot statistics are: 94.0% (568/604) of all residues were in favored (98%) regions residues in additional allowed regions 99.0% (598/604) of all residues were in allowed (>99.8%) regions. The figure was generated using RAMPAGE web server (http://mordred.bioc.cam.ac.uk/).

Finally, the recognition of artefacts and errors in experimental and theoretical structures remains a problem in the field of structure modeling. A structural comparison of Cry1Aa toxin with the theoretical model of the Cry1Ab15 protein indicates correspondence with the general model for a Cry protein the superimposed backbone traces showed low RMS deviations (Figure 5). The comparison between the overall energy of developed structure and those of experimentally determined structures in PROSA database validated the developed model as folded near to experimentally determined, natural structures (Figure 6) while the Ramachandran plot analysis (Figure 7) supported the above conclusions by showing that most of the residue (98%) has *φ* and *ψ* angles in the core- and allowed-regions, except for nine residues which qualified for outlier region. Most bond lengths, bond angles, and torsion angles were in the range of values expected for a naturally folded protein (Figure 7).

## 4. Conclusions

In conclusion, evidence presented here, based on the identification of structural equivalent residues of Cry1Aa in Cry1Ab15 toxin through homology modeling, indicates that due to the high amino acid homology between these two toxins, they do share a common three-dimensional structure. Cry1Aa and Cry1Ab15 contain the most variable regions in the loops of domain II, which is responsible for the specificity of these toxins. Structural comparison indicates a correspondence to the general model for a Cry protein (an *α* + *β* structure with three domains) and few of the differences present are the presence of 175 *β*0 and *α*3 the absence of *α*7b, *β*1a, *α*10a, *α*10b, *β*12, and *α*11a while *α*9 is located spatially downstream. This is the first model of a Cry1Ab15 protein and its importance can be perceived since the members of this group of toxins are potentially important entomopathogenic candidates.

## Figures and Tables

**Table 1:** The comparison among three-domain structural components of Cry1Aa and Cry1Ab15 toxin molecules.

Domain I			Domain II			Domain III		
	Cry1Aa	Cry1Ab15		Cry1Aa	Cry1Ab15		Cry1Aa	Cry1Ab15
*β*0	—	Thr31-Tyr33	*α*8a	Pro271-Glu274	Pro271-Asn275	*α*11a	Leu475-Lys477	—
*α*1	Pro35-Ser48	Pro35-Ser48	*α*8b	Ala284-Gln289	Ser283-Ser290	*β*13a	Ser486-Val488	Ser487-Val489
*α*2a	Aln54-Ile63	Aln54-Trp65	*β*2	Asp298-His310	Ile299-His310	*β*13b	Ile498-Arg501	Ile499-Arg502
*α*2b	Pro70-Ile84	Pro70-Ile84	*β*3	Phe313-Trp316	Glu313-Ser324	*β*14	Gly505-Asn513	Gly506-Asn514
*α*3	Glu90-Ala119	Glu90-Ala119	*β*4	Gly318-Pro325	Tyr359-Arg368	*β*15	Tyr522-Ser530	Tyr523-Ala530
*α*4	Pro124-Leu148	Pro124-Leu148	*α*9	Val326-Phe328	*Ser409-Glu412	*β*16	Leu534-Ile540	Leu534-Ile541
*α*5	Gln154-Trp182	Val155-Trp182	*β*5	Val348-Ser351	Leu380-Tyr390	*β*17	Arg543-Phe550	Arg544-Phe551
*α*6	Ala186-Val218	Ala186-Val218	*β*6	Ile357-Arg367	Ala399-Tyr401	*α*12a	Ser562-Ser564	Ser563-Ser565
*α*7a	Ser223-Thr239	Ser223-Tyr250	*β*7	Leu380-Leu383	Thr406-Asp408	*β*18	Arg566-Gly569	Arg567-Phe571
*α*7b	Leu241-Tyr250	—	*β*8	Gly385-Phe390	His428-Arg437	*β*19	Ser580-His588	Ser581-His589
*β*1a	Glu266-Thr269	—	*β*9	Thr400-Tyr402	Ile447-His457	*β*20	Val596-Pro605	Val597-Pro606
			*α*10a	Ser410-Asp412	—	*α*13	—	Phe611-Ala623
			*α*10b	Pro423-Gly426	—			
			*β*10	His429-Val434	Gln473-Pro475			
			*β*11	Phe452-His456	Thr480-Leu482			
			*β*12	Thr471-Pro474	—			

—: similar component not present. *Components in italics are spatially present at downstream sites.
